# Effect of a lay counselor-delivered integrated maternal mental health and early childhood development group-based intervention in Northern Ghana: a cluster-randomized controlled trial

**DOI:** 10.1017/gmh.2021.15

**Published:** 2021-05-26

**Authors:** Joy Noel Baumgartner, Mohammed Ali, John A. Gallis, Margaret Lillie, Raymond Owusu, Safiyatu Abubakr-Bibilazu, Haliq Adam, Raymond Aborigo, Elena McEwan, Yunji Zhou, Eunsoo Timothy Kim, Jessica Mackness, John Koku Awoonor Williams, John Hembling

**Affiliations:** 1 University of North Carolina at Chapel Hill, 325 Pittsboro Street, Chapel Hill, NC 27516, USA; 2 Duke Global Health Institute, Duke University, Durham, NC, USA; 3 Catholic Relief Services Country Office, Tamale, Ghana; 4 Duke Department of Biostatistics and Bioinformatics, Durham, NC, USA; 5 Navrongo Health Research Centre, Navrongo, Ghana; 6 Catholic Relief Services Head Quarters, Baltimore, MD, USA; 7 Ghana Health Service, Accra, Ghana

**Keywords:** Early childhood development, Ghana, infant mental health, maternal depression, socio-emotional health

## Abstract

**Background:**

Caregiver mental health is linked to early childhood development, yet more robust evidence of community-based interventions to prevent maternal depression and optimize socio-emotional development of young children is needed. Objectives of this cluster-randomized controlled trial (cRCT), based in Northern Ghana, are to assess the impact of the lay counselor-delivered, group-based Integrated Mothers and Babies Course and Early Childhood Development (iMBC/ECD) program on (1) the mental health of mothers of children under age 2; and (2) the socio-emotional development of their children.

**Methods:**

This cRCT randomized 32 women's groups – 16 received iMBC/ECD content (intervention) and 16 received general health education content (control). Surveys were administered at baseline, immediate post-intervention, and 8-month post-intervention. The primary outcome was maternal depression [Patient Health Questionnaire (PHQ-9)], and the secondary outcome was child's socio-emotional development [Ages and Stages Questionnaire: Social Emotional (ASQ:SE-2)]. Qualitative interviews with 33 stakeholders were also conducted.

**Results:**

In total, 374 participants were enrolled at baseline while pregnant with the index child, 19% endorsing moderate/severe depression. Of these, 266 (71.1%) completed the 8-month post-intervention survey (~19 months post-baseline). There were no significant effects of iMBC/ECD on PHQ-9 and ASQ:SE-2 scores. However, results favored the intervention arm in most cases. iMBC participants were highly satisfied with the program but qualitative feedback from stakeholders indicated some implementation challenges.

**Conclusions:**

This real-world evaluation had null findings; however, post-intervention depression levels were very low in both arms (3%). Future research should examine the potential impact of women's groups on postpartum mental health more broadly with varying content.

## Background

The Nurturing Care Framework and recent World Health Organization (WHO) guidance on improving early childhood development (ECD) clearly recognize the critical role of positive maternal mental health for optimal ECD and recommend that interventions integrate support for caregiver mental health with early childhood health and development services (World Health Organization *et al*., 2018; World Health Organization, 2020). Maternal psychological disorders during pregnancy and the postnatal period have been identified as a key risk factor for poor child development outcomes in growth, cognition, school achievement, and overall child health in low- and middle-income countries (LMICs) (Rahman *et al*., 2004; Sawyer *et al*., 2010; Surkan *et al*., 2011; Albanese *et al*., 2019; Claessens *et al*., 2015). Likewise, there is evidence that maternal self-efficacy is affected by depression, and lower self-efficacy predicts poor child social-emotional and cognitive outcomes (Albanese *et al*., 2019).

A critical protective factor in the first years of life is positive, supportive, developmentally appropriate interactions with parents and/or caregivers (Aboud and Yousafzai, 2015). There is growing evidence to suggest that community-based interventions, delivered by trained community volunteers, can have a significant impact on perinatal depression and/or child development outcomes (Chowdhary *et al*., 2014; Singla *et al*., 2015). For example, in rural Uganda, a cluster-randomized controlled trial (cRCT) revealed that a community-based parenting intervention reduced maternal depressive symptoms and improved child development outcomes in cognition and receptive language outcomes compared to a control group (Singla *et al*., 2015). A systematic review of nine studies conducted in LMICs revealed that interventions delivered by non-professional health workers were effective in treating perinatal depression (Chowdhary *et al*., 2014).

Ghana has endorsed and prioritized the Nurturing Care Framework for advancing ECD which includes a focus on parental mental health to support responsive caregiving (UNICEF and Countdown to 2030, 2019). There is global and Ghana-specific evidence that maternal depression negatively affects parenting practices, including under-stimulation, and child health outcomes including socio-emotional health, yet the evidence base for real-world, scaled interventions applicable to rural populations is still limited (Wemakor and Mensah, 2016; Huang *et al*., 2018; Jeong *et al*., 2018; Madlala and Kassier, 2018).

The goal of this study is to evaluate the impact of a group-based, cognitive-behavioral therapy intervention for women delivered during pregnancy and the postpartum period. The two primary aims are to assess the impact of the Integrated Mothers and Babies Course and Early Childhood Development (iMBC/ECD) intervention on (1) the mental health of mothers of children under age 2 living in rural northern Ghana; and (2) the socio-emotional development of their children.

## Methods

### Intervention

The Integrated Mothers and Babies Course (iMBC) is an evidence-based intervention for preventing postpartum depression that has been tested with low-income English- and Spanish-speaking pregnant women at high risk for depression in the USA and adapted for use in low-resource settings (Le *et al*., 2010, 2015; Le, 2018). The iMBC content is based on the principles of cognitive-behavioral therapy and attachment theory. The new iMBC/ECD curriculum was designed with the aim of supporting pregnant women and mothers with children under 2 years of age to manage daily stressors effectively, promote early stimulation behaviors (ESB) and bonding to support child development, and decrease future risk of depression. The theoretical basis for iMBC is that improved mood coupled with ECD knowledge and skills acquired during group sessions will lead to greater parenting self-efficacy, positive parenting practices, and ultimately improved child development.

In Ghana, the iMBC/ECD intervention was implemented in the context of the Rural Emergency Health Service and Transport (REST II) project led by Catholic Relief Services (CRS), an international, non-governmental organization working in Northern Ghana in collaboration with the Ghana Health Service (GHS). REST II combines both facility and community-based approaches across 10 districts to improve Maternal, Newborn, Child Health, and Nutrition (MNCHN) practices and pre/postnatal health service utilization. A key activity of REST II is Community Pregnancy Surveillance and Targeted Education Sessions (C-PrES). Routine C-PrES is delivered via educational group sessions that promote the adoption of key MNCHN behaviors (e.g. newborn care, exclusive breastfeeding, etc.) among pregnant women and mothers of children under age 2 with content endorsed by the GHS. C-PrES groups are composed of ~20–25 women from the same community. iMBC/ECD was delivered via C-PrES groups and implemented by ‘model mothers’ who were women from the communities and supervised by GHS community health officers and CRS REST II field staff. The model mothers were jointly identified by CRS and GHS staff as women who were pregnant or had a child under age 2 and who exhibited what they considered healthy MNCHN-related behaviors. They were also supposed to be literate in the local language. The model mothers, alongside GHS staff nurses, received a 1-week training in July 2018 and a 3-day refresher training in November 2018 on iMBC delivered by masters-level CRS staff who had themselves been trained by one of the iMBC original developers, a clinical psychologist based in the USA and a master iMBC trainer from Kenya. Weekly supervision by GHS nurses was also provided to the model mothers to review and practice content.

iMBC/ECD was delivered during 14 group sessions (~1 h) administered every 2 weeks over a 7-month period. Regular C-PrES groups met with the same frequency. The modules guided mothers to describe and manage their internal and external realities through the application of select coping skills. The iMBC curriculum also integrated key ECD messages (e.g. importance of antenatal care, breastfeeding and nutrition, seeking timely care for sick child). To support behavior change, home visits were conducted once a month to check on mothers' mood, assess uptake of negotiated ECD behaviors, and encourage husbands and grandmothers to support mothers' participation in the project. After the 14 sessions, mothers were invited to continue to receiving age-appropriate ECD integrated messages along with five additional iMBC booster sessions.

The ECD content within both the integrated iMBC/ECD intervention and the C-PrES control groups aligned with early stimulation content appropriate during pregnancy and the first 2 years of a child's life, namely play and communication activities as highlighted by UNICEF's Care for Child Development (WHO and UNICEF, 2012).

### Study design

This study is a longitudinal cRCT of 32 community-based women's groups (ClinicalTrials.gov # NCT03665246) to evaluate the impact of the iMBC/ECD intervention that was administered by CRS.

The study collected data at four time points. Baseline data (August 2018) were collected when participants were pregnant and the timing was intended to be right before the beginning of the groups; however, there was about a 3-month delay between baseline data collection and the start of the program, so an additional ‘mini’ survey focused on mental health measures was added (December 2018) to ensure no dramatic differences in mental health status at baseline/pre-intervention. The first follow-up data collection period (follow-up 1) was immediate post-intervention (July 2019) and the second follow-up (follow-up 2) was 8-months post-intervention (February 2020).

### Sample size

We targeted recruitment of 16 clusters per arm (32 total clusters) assuming a standardized effect size on the primary outcomes of approximately half a standard deviation (i.e. 0.5), and an ICC of 0.04 based on preliminary data from a similar study in Cameroon (NCT03195036). Assuming the possibility of one cluster dropping out, we had 91.6% power to detect this effect size at an *α* of 0.05, with an average of 10 total participants per group and a large coefficient of variation of cluster sizes of 0.850. After recruitment, we had 374 total participants across 32 clusters, with an average cluster size of 11.7 and a coefficient of variation of cluster sizes of 0.79.

### Randomization and masking

We performed stratified, constrained randomization (Raab and Butcher, 2001). The randomization was stratified by district and constrained by 2018 population estimates, distance to the nearest health facility, and type of health facility (see Table 2). Constrained randomization was implemented using the cvcrand Stata package by the US-based team (Gallis *et al*., 2018). Both the intervention and control groups could have been identifiable to participants and/or the research assistants (RAs). The analysis team was not masked; however, a statistical analysis plan was created prior to follow-up 2 data collection.

### Study participants and study setting

All of the participant women were enrolled in the group-based C-PrES program, waiting for the program to begin. Each C-PrES group was randomized to receive one of two programs – the intervention groups were exposed to maternal mental health content with integrated ECD messages (iMBC/ECD) and the control group was exposed to the usual basic maternal and child education messages (e.g. timely antenatal/postnatal care, nutrition/feeding, childhood illnesses, etc.). Therefore, the study had 16 iMBC/ECD groups and 16 C-PrES only groups.

To participate in this study conducted from August 2018 to February 2020, eligible participants had to be registered in a C-PrES group, 16 years or older, currently pregnant at baseline, and planning to maintain residence in the community for at least 6 months (duration of the program). The study was conducted in 32 communities (clusters) in two districts in Northern Ghana: West Mamprusi District in the North East Region where Mampruli is spoken, and Nabdam District in the Upper East Region where Nabt is spoken. Each community had one available women's group in which all pregnant women in the community could enroll, resulting in 32 women's groups across the study participants. Additional details on data collection procedures have been previously published (Lillie *et al*., 2020; Mackness *et al*., 2021).

### Data collectors

Both quantitative and qualitative data were collected by a total of 24 RAs who received 1 full week of training prior to every data collection time point (3 trainings), which included ethics, community-based pre-testing activities, orientation to tablet-based CommCare data collection, interviewing skills, and procedures for referrals as needed (e.g. mental health, domestic violence, child undernutrition). The RAs were literate in English, spoke the local languages and were recruited from the communities.

### Primary outcome measures

For our primary maternal mental health measure, we used the Patient Health Questionnaire (PHQ-9), a depression screener that has been previously validated and recommended for use in Ghana, though not in the Mampruli or Nabt languages (Kroenke *et al*., 2001; Kroenke and Spitzer, 2002; Weobong *et al*., 2009). The nine items of the PHQ-9 are summed for a score between 0 and 27. The PHQ-9 underwent a forward and back translation process for Mampruili and Nabt, with professional translators overseeing a group consensus process to address any discrepancies before arriving at the final translations. The Cronbach's *α* for the PHQ-9 was 0.82 at baseline, 0.88 at first follow-up and 0.84 at the last follow-up indicating good internal consistency.

For our primary child socio-emotional development measure, we used the Ages and Stages Questionnaire Social Emotional (ASQ:SE-2) (Squires *et al*., 2015). The ASQ:SE-2 is a parent-reported survey with multiple age intervals of varying numbers of questions to identify young children at risk for social emotional difficulties including screens for self-regulation compliance, communication, adaptive functioning, autonomy, affect, and interaction with people. This study used the 2-month (1–2 months 30 days), 6-month (3–8 months 30 days), 12-month (9–14 months 30 days), 18-month (15–20 months 30 days), and 24-month (21–26 months 30 days) intervals. Parent response options were ‘often or always’, ‘sometimes’, or ‘rarely or never’ to each question and then a yes or no question if the parent has a concern about the specified behavior. The higher the score, the higher the risk of social emotional difficulties. The ASQ:SE-2 was forward translated into Mampruli and Nabt and the RAs reviewed and revised the translations as a team during training. In order to compare study children across age ranges (at follow-up 2, three age interval ASQ:SE-2 forms were relevant), we took the average item score for each child at each time point (FAQ – Scoring ASQ:SE-2 [online] 2019). The Cronbach's *α* for the ASQ:SE-2 at the last follow-up was 0.71 for the 6-month version, 0.68 for the 12-month version, and 0.65 for the 18-month version indicating adequate internal consistency.

### Covariates

Socioeconomic status was assessed using the Ghana Equity Tool which uses data from the DHS to create a list of assets that may be important indicators of household wealth (Ghana Equity Tool [online] 2016). We added two additional questions about assets that the local data collection team in Ghana thought were important assets that may determine wealth (Does your household have a satellite dish? Does anyone in your household use mobile money?). To make one single SES variable, we generated a previously devised asset index using the polychoric correlation principal component analysis approach to create five wealth quintiles based on the Ghana Equity Tool and the two additional questions listed above (Kolenikov and Angeles, 2009; Maselko *et al*., 2018). Those items in the equity tool that did not show much variability (>90% owned or did not own asset) were excluded from the creation of the asset index.

The Household Hunger Scale (HHS) was used to assess food insecurity (Ballard *et al*., 2011). HHS is a six-question scale [three topical questions (no food in the house, hunger when going to sleep, lack of food in 24 h) which are each followed by questions asking the frequency of occurrence] which is summed to create a score from 0 to 6 with higher scores indicating higher levels of food insecurity. We used the standard categorical variable indicating little to no hunger (0–1 point), moderate to severe hunger (2–3 points), and severe hunger (4–6 points).

Maternal early stimulation behaviors (ESB) during pregnancy were assessed with four questions: during this pregnancy, how often do you talk softly to him/her and touch belly, sing songs to him/her, tell him/her about his/her family, and dance to music. Each ESB was scored never (0 points), rarely (1 point), sometimes (2 points), frequently (3 points). The maternal–fetal relationship, which focuses on interactions and feelings the expectant mother has toward her developing fetus, is one of the first opportunities in ECD to create a nurturing relationship between caregiver and baby and research has shown that touching and talking to the belly is widely recognized and measured as an indicator of maternal–fetal attachment (Cranley, 1981; Muller and Mercer, 1993). Only ESB during pregnancy was included in analyses as this was needed for adjustments for balance imbalances. ESB at older ages are assumed at random across the treatment and control arms.

Physical, sexual, and emotional intimate partner violence during the past 12 months were assessed using standard items from the 2008 Ghana Demographic and Health Survey only for those participants who had a partner in the past 12 months (Ghana Statistical Service *et al*., 2009). Domains were specified using the Guide to DHS Statistics (emotional IPV, physical IPV, sexual IPV) (Croft *et al*., 2018). For analysis, each domain was represented using a binary variable indicating if the participant endorsed at least one item within the domain. Physical and sexual IPV were collapsed into one domain for analysis because these were the IPV domains that required a referral during data collection for our study population.

Additional questions included the woman's age, relationship status, education, number of lifetime pregnancies, and self-reported physical health. For participants in the iMBC groups, there were also questions about their experience with the overall program and specific content.

### Data management and analyses

Data were collected via the CommCare platform and were uploaded and synced with the main database and processed and analyzed using Stata/SE version 16.1 (StataCorp, College Station, TX, USA). Baseline characteristics of recruited mothers and cluster-level characteristics were reported by study arm. Continuous variables were summarized by means and standard deviations (s.d.), and categorical variables by counts and percentages. The primary analyses were designed as intent-to-treat. The primary mother outcome is PHQ-9 score at the 8-month post-intervention follow-up (follow-up 2) and the primary child outcome is the mean ASQ:SE-2 score at follow-up 2.

The two primary outcomes were analyzed using linear mixed-effects models so that all comparisons of interest could be estimated from the same model. All mixed models included C-PrES group as a random intercept to account for the clustered study design. In addition, we included a random intercept and a random slope for repeated measures at the follow-up time points for each participant for the primary endpoints. Restricted maximum likelihood estimation was used in all models.

In the primary statistical models, we adjusted for the four variables upon which the randomization was stratified and constrained: district, 2018 population estimates, distance to the nearest health facility, and health facility type (Table 2). In addition, we summarized baseline characteristics by intervention arm and by missing at follow-up. If we identified baseline variables associated with loss-to-follow-up or imbalanced at baseline, we adjusted for these variables as sensitivity analyses. We considered a variable to be imbalanced by loss to follow-up or by intervention arm if the corresponding *p* value is <0.10.

For all outcomes, we additionally reported a *p* value comparing intervention and control using a clustered permutation test adjusted for the cluster-level variables on which the randomization was stratified and constrained, as is appropriate after constrained randomization, using the cptest program in Stata/SE.16.1 (Gallis *et al*., 2018). In addition, we checked the robustness of the individual-level regressions using cluster-level analysis (Hayes and Moulton, 2017).

Exploratory analyses were conducted to examine if the effect of the intervention on the primary outcomes differed by the level of potential effect modifiers listed in online Supplementary Figs S1–S4. Also, 72% of the participants (*n* = 136) in the intervention arm reported attending more than half or all of the sessions, excluding 28 participants lost to follow-up and three participants with unknown attendance data. We performed separate ‘per-protocol’ analyses on the primary outcomes, wherein we performed the same analyses as described above but included those in intervention who attended up to ‘about half’ of the sessions in the control group. Additional exploratory analysis examined the primary outcomes by baseline mental health status (none/mild and moderate/severe depression).

**Fig. 1.**
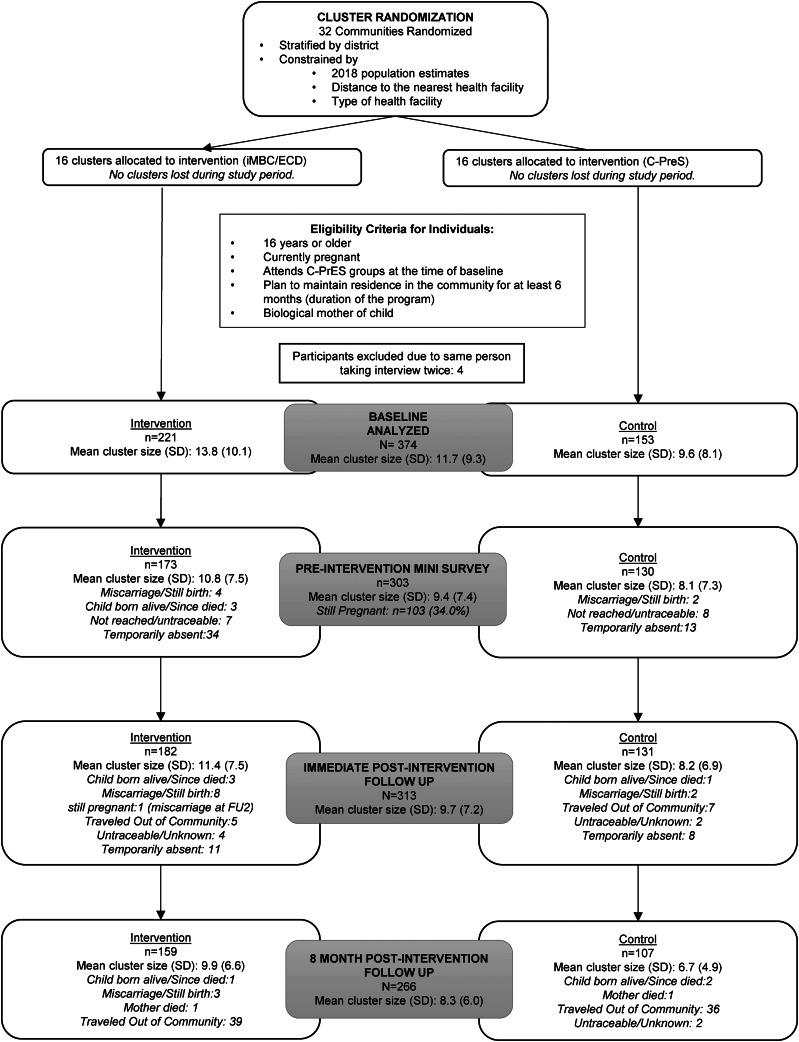
CONSORT Diagram.

Statistical analyses were conducted and reported according to the CONSORT guidelines (Fig. 1) and the CONSORT extension for Cluster Trials (Schulz *et al*., 2010; Campbell *et al*., 2012).

### Qualitative data collection and analysis

To gain perspective on the implementation and acceptability of iMBC/ECD, 33 semi-structured interviews (SSIs) with key informants and two focus group discussions (FGDs) were conducted during the immediate post-intervention period in the iMBC communities only. SSIs were conducted with three district-level leaders, two CRS field officers, 10 GHS iMBC supervisors, nine model mothers, nine iMBC group participants, and two FGDs were conducted with 10 husbands of iMBC group participants. SSIs (~45 min long) and FGDs (~1 h long) were conducted in either Mampruli or Nabt as appropriate. SSI/FGD topic domains included key informant's role in implementing and/or participating in iMBC, what they liked or disliked about the program, challenges and opportunities for future implementation/participation. Interviews were recorded and transcribed into English. Rapid content analysis was conducted using an analysis matrix in Microsoft Excel.

### Ethical approvals

Ethical approval was received from the Duke University Campus IRB (# 2019-0020) and the Navrongo Health Research Center in Ghana (# NHRCIRB314). All participants (clinical trial cohort, individual interviews, and FGDs) signed a written informed consent form, or, if they were illiterate, were read the consent form and provided their fingerprint with a witness signature. Per best practices for conducting research on mental health and gender-based violence and GHS recommendations, all participants who endorsed suicidal ideation during the survey were offered a mental health referral and all participants who reported physical or sexual violence in the last 12 months were offered a referral for domestic violence social services. [Note: At baseline, we only documented if the referral was offered. For follow-up surveys, we started documenting if referrals were accepted as participants had the right to decline the referral. Starting at the follow-up time points, we documented acceptance of mental health and IPV referrals. For mental health, 100% (27 of *n* = 27 at FUP 1) and 91% (10 of *n* = 11 at FUP 2) accepted a referral to psychiatric services. For IPV, 48% (19 of *n* = 40 at FUP1) and 26% (9 of *n* = 35 at FUP2) accepted a referral to social services.]

## Results

There were 374 participants in the study at baseline, from 32 C-PrES groups. Of these, 303 (81%), 313 (83.7%), and 266 (71.1%) participants were followed up at the mini-survey, immediate post-intervention survey (follow-up 1), and the 8-month post-intervention survey (follow-up 2) time points, respectively (Fig. 1). At baseline, the average age of the women was 27 years old, with almost half (49%) having never received formal education and 39% of the women indicating that they experienced physical and/or sexual violence from an intimate partner in the past year. Further baseline individual characteristics including *p* values (unclustered) to check for baseline imbalance (defined as *p* value <0.10) are presented in Table 1 (Bolzern *et al*., 2019).

**Table 1.** Baseline characteristics of the intent-to-treat population in a cluster-randomized controlled trial of iMBC program among women in Northern Ghana, 2018

	Control	Intervention	Total	
	(*N* = 153)	(*N* = 221)	(*N* = 374)	*p* value
Age in years				0.999
Mean (s.d.)	26.95 (6.53)	26.95 (6.99)	26.95 (6.80)	
Min, Max	16.0, 45.0	16.0, 50.0	16.0, 50.0	
Number of pregnancies				0.560
Mean (s.d.)	3.26 (1.78)	3.38 (2.04)	3.33 (1.93)	
Min, Max	1.0, 9.0	1.0, 9.0	1.0, 9.0	
SES asset index (quintiles)				0.022
Lowest quintile	33 (21.6%)	40 (18.4%)	73 (19.7%)	
Second quintile	40 (26.1%)	33 (15.2%)	73 (19.7%)	
Middle quintile	28 (18.3%)	48 (22.1%)	76 (20.5%)	
Fourth quintile	29 (19.0%)	40 (18.4%)	69 (18.6%)	
Highest quintile	23 (15.0%)	56 (25.8%)	79 (21.4%)	
Household hunger in past 30 days				0.161
None/little	114 (74.5%)	159 (71.9%)	273 (73.0%)	
Moderate	34 (22.2%)	60 (27.1%)	94 (25.1%)	
Severe	5 (3.3%)	2 (0.9%)	7 (1.9%)	
Highest level of school attended				0.141
No education	74 (48.4%)	108 (48.9%)	182 (48.7%)	
Primary	39 (25.5%)	51 (23.1%)	90 (24.1%)	
Post-primary	23 (15.0%)	49 (22.2%)	72 (19.3%)	
Secondary/A level	14 (9.2%)	13 (5.9%)	27 (7.2%)	
College/university	3 (2.0%)	0 (0.0%)	3 (0.8%)	
Self-reported physical health				0.269
Excellent	18 (11.8%)	17 (7.7%)	35 (9.4%)	
Very good	64 (41.8%)	82 (37.1%)	146 (39.0%)	
Good	53 (34.6%)	82 (37.1%)	135 (36.1%)	
Fair	17 (11.1%)	35 (15.8%)	52 (13.9%)	
Poor	1 (0.7%)	5 (2.3%)	6 (1.6%)	
Relationship status				0.094
Married, living with husband	140 (91.5%)	194 (89.0%)	334 (90.0%)	
Married, not living with husband	7 (4.6%)	19 (8.7%)	26 (7.0%)	
Living with partner, not married	2 (1.3%)	0 (0.0%)	2 (0.5%)	
Has partner/not married or living together	1 (0.7%)	4 (1.8%)	5 (1.3%)	
No current partner	3 (2.0%)	1 (0.5%)	4 (1.1%)	
Intimate partner physical and/or sexual violence in past 12 months				0.025
No	103 (68.2%)	117 (56.5%)	220 (61.5%)	
Yes	48 (31.8%)	90 (43.5%)	138 (38.5%)	
Intimate partner emotional violence in past 12 months				0.015
No	95 (62.9%)	104 (50.0%)	199 (55.4%)	
Yes	56 (37.1%)	104 (50.0%)	160 (44.6%)	
Early stimulation behaviors during pregnancy total score (4 items)				0.004
Mean (s.d.)	4.03 (1.52)	4.54 (1.73)	4.33 (1.66)	
Min, Max	2.7, 8.7	2.7, 10.7	2.7, 10.7	
*Early stimulation behaviors by mother during pregnancy: Do you…*				
(1) Talk softly to him/her and touch belly?				0.001
Never	83 (54.2%)	75 (33.9%)	158 (42.2%)	
Rarely	18 (11.8%)	25 (11.3%)	43 (11.5%)	
Sometimes	39 (25.5%)	93 (42.1%)	132 (35.3%)	
Frequently	13 (8.5%)	28 (12.7%)	41 (11.0%)	
(2) Sing songs to him/her?				0.713
Never	111 (72.5%)	156 (70.6%)	267 (71.4%)	
Rarely	16 (10.5%)	24 (10.9%)	40 (10.7%	
Sometimes	24 (15.7%)	34 (15.4%)	58 (15.5%)	
Frequently	2 (1.3%)	7 (3.2%)	9 (2.4%)	
(3) Tell him/her about his/her family?				0.417
Never	122 (79.7%)	162 (73.3%)	284 (75.9%)	
Rarely	14 (9.2%)	22 (10.0%)	36 (9.6%)	
Sometimes	16 (10.5%)	33 (14.9%)	49 (13.1%)	
Frequently	1 (0.7%)	4 (1.8%)	5 (1.3%)	
(4) Dance to music?				0.187
Never	107 (69.9%)	133 (60.2%)	240 (64.2%)	
Rarely	15 (9.8%)	24 (10.9%)	39 (10.4%)	
Sometimes	30 (19.6%)	59 (26.7%)	89 (23.8%)	
Frequently	1 (0.7%)	5 (2.3%)	6 (1.6%)	
*Social support: In the last month, how much assistance did you receive from*				
Husband/partner				0.149
Never/insufficient	75 (49.7%)	126 (57.3%)	201 (54.2%)	
Sufficient	76 (50.3%)	94 (42.7%)	170 (45.8%)	
Male relatives				0.190
Never/insufficient	113 (73.9%)	176 (79.6%)	289 (77.3%)	
Sufficient	40 (26.1%)	45 (20.4%)	85 (22.7%)	
Female relatives				0.100
Never/insufficient	89 (58.2%)	147 (66.5%)	236 (63.1%)	
Sufficient	64 (41.8%)	74 (33.5%)	138 (36.9%)	
Female friends				0.870
Never/insufficient	127 (83.0%)	182 (82.4%)	309 (82.6%)	
Sufficient	26 (17.0%)	39 (17.6%)	65 (17.4%)	
*Maternal mental health*				
PHQ-9 symptom severity				0.033
None/minimal (0–4)	69 (45.1%)	70 (31.7%)	139 (37.2%)	
Mild (5–9)	62 (40.5%)	99 (44.8%)	161 (43.0%)	
Moderate (10–14)	19 (12.4%)	43 (19.5%)	62 (16.6%)	
Moderately severe (15–19)	3 (2.0%)	9 (4.1%)	12 (3.2%)	
PHQ-9 score				0.003
Mean (s.d.)	5.54 (3.82)	6.81 (4.21)	6.29 (4.10)	
Min, Max	0.0, 16.0	0.0, 16.0	0.0, 16.0	

The following variables, ESB four-item score during pregnancy, PHQ-9 score, asset SES index, relationship status, and history of physical/sexual/emotional violence, were all imbalanced (*p* < 0.10) at baseline between intervention and control groups and thus necessitated sensitivity analyses (Table 1). In addition, variables were examined for differential missingness at each time point and the highest level of school attended was identified to differ by missing status at follow-up 2 (data not shown), which also required sensitivity analysis. *A priori* cluster-level characteristics of interest are summarized in Table 2.

**Table 2.** Cluster-level characteristics for study communities (*N* = 32 clusters)

	Control	Intervention	Total
	(*N* = 16)	(*N* = 16)	(*N* = 32)
District			
Nabdam	8 (50.0%)	8 (50.0%)	16 (50.0%)
West Mamprusi	8 (50.0%)	8 (50.0%)	16 (50.0%)
2018 population estimates			
Mean (s.d.)	788.19 (364.05)	1177.06 (878.92)	982.63 (690.61)
Min, Max	362.0, 1473.0	217.0, 2941.0	217.0, 2941.0
Distance to nearest health facility (km)			
Mean (s.d.)	7.31 (3.40)	7.31 (4.19)	7.31 (3.75)
Min, Max	3.0, 16.0	0.0, 18.0	0.0, 18.0
Type of health facility			
CHPS compound	11 (68.8%)	12 (75.0%)	23 (71.9%)
Health center	4 (25.0%)	3 (18.8%)	7 (21.9%)
Hospital	1 (6.3%)	1 (6.3%)	2 (6.3%)

Regression results are presented in Table 3. There were no significant effects of the iMBC intervention on PHQ-9 and ASQ:SE-2 scores. However, the results favored the intervention arm in most cases. At baseline, the predicted average PHQ-9 score in the treatment arm was 6.8 (6.2, 7.4). In the control arm, the predicted average PHQ-9 score was 5.5 (4.9, 6.1). At 8-month post-intervention, we observed a slightly larger decrease in PHQ-9 score from baseline in the treatment arm than in the control arm [estimate (95% CI) −0.8 (−1.9 to 0.3)]. There was no significant effect of intervention on the average ASQ:SE-2 mean score at 8-month post-intervention [estimate (95% CI) 0.02 (−0.5 to 0.6)].

**Table 3.** Estimated intervention effects for maternal mental health and socio-emotional development of children in Northern Ghana

Maternal mental health	Predicted mean PHQ-9 (intervention)	Predicted mean PHQ-9 (control)	Predicted mean change in PHQ-9 from baseline, (intervention)	Predicted mean change in PHQ-9 from baseline, (control)	Predicted mean difference in change from baseline, intervention *v*. control
Baseline	6.8 (6.2, 7.4)	5.5 (4.9, 6.1)			
Mini survey	6.0 (5.3, 6.7)	4.9 (4.2, 5.6)	−0.8 (−1.4, −0.2)	−0.6 (−1.3, 0.1)	−0.2 (−1.1, 0.7)
Follow-up 1	2.8 (2.1, 3.5)	1.6 (0.9, 2.3)	−4.0 (−4.6, −3.4)	−3.9 (−4.6, −3.2)	−0.1 (−1.1, 0.9)
Follow-up 2	1.9 (1.1, 2.7)	1.5 (0.7, 2.3)	−4.9 (−5.6, −4.2)	−4.1 (−4.9, −3.2)	−0.8 (−1.9, 0.3)
Child socio-emotional development	Predicted mean ASQ:SE-2 (intervention)	Predicted mean ASQ:SE-2 (control)			Predicted mean difference, intervention *v*. control
Follow-up 1	3.2 (2.5, 3.9)	2.7 (2.1, 3.3)			0.3 (−0.2, 0.9)
Follow-up 2	2.5 (1.8, 3.2)	2.4 (1.8, 3.0)			0.02 (−0.5, 0.6)

Online Supplementary Table S1 displays the primary outcome variable summaries by treatment arm. Results of sensitivity analyses (adjusting for baseline imbalances as noted in Table 1 and the differential loss to follow-up by education level) were similar to the estimated effects from the primary analyses. No significant effects were detected using cluster-level analysis and clustered permutation test. No significant effect modifiers were identified (online Supplementary Figs S1–S4).

Per-protocol analyses for treatment exposure provided similar estimates of the treatment effect at the primary time point, follow-up 2 (online Supplementary Table S2: continuous regression results for PHQ-9 score and ASQ-SE by high attendance intervention group *v.* low attendance + control group). High attendance included those who reported attending all or more than half sessions (61.5%). Those who reported attending half, less than half the sessions, no sessions, or who were unsure of how many sessions they attended were grouped with the control participants for a ‘no exposure’ group (see online Supplementary Table S3 for attendance data). Exploratory analyses examining changes in depression among only those who had moderate or severe symptoms of depression at baseline also revealed no difference between groups over time.

Among participants in the intervention arm (iMBC/ECD groups), 93% reported being ‘very much’ or ‘extremely’ satisfied with the program, and 99% said they would recommend the program to a friend. Intervention participants were generally positive about the program indicating they highly valued some program components such as how to change their moods and thoughts, how to better communicate with the people in their lives (families, husband, mother-in-law), and how to care for their baby.

Qualitative SSI data from iMBC participants and implementers were also largely positive of the program overall, with comments that women were able to participate in the program due to familial support and that refreshments could help with attendance. Participants recalled key messages from both iMBC and ECD content (e.g. pleasant activities to improve mood and exclusive breastfeeding) but also indicated that some iMBC content was challenging such as ‘thought interruption’ and having scheduled ‘worry time’.

Focus groups with husbands/partners revealed they were broadly supportive and interested in engaging in similar programming for themselves, noting that the biggest parental stressor was lack of money for food, health services, and education. They also highlighted learning from their partners how parental good moods affect child outcomes and women sharing what makes them happy/sad helps with partner communication.

Qualitative data from government stakeholders and program implementers revealed high levels of support for the continuation of the iMBC/ECD program but also implementation challenges related to the literacy of Model Mothers (difficulty delivering content), interpretation of course content into local languages, and attendance of participants. Illustrative quotes from the qualitative data can be found in online Supplementary Table S4.

## Discussion

In this trial, we found no difference between the iMBC groups (maternal mental health/ECD intervention) and the ECD only controls over time on the main outcome variables (maternal depression and child social-emotional development). Our study revealed that a substantial portion of participants across both groups were experiencing moderate to severe symptoms of depression (PHQ-9 ⩾ 10) particularly during pregnancy (19.8%, *n* = 74), but less so during the early postpartum period (4.79%, follow-up 1, *n* = 15) and the extended postpartum period (3.01%, follow-up 2, *n* = 8).

However, our results make several important contributions to the evaluation literature on addressing the socio-emotional development of young children in tandem with maternal mental health as a global strategy for ensuring children reach their full potential and caregivers have the internal resources they need to care for their families (World Health Organization, 2020). First, while depression symptoms decreased over time in both study arms, the prevalence of perinatal depression more broadly reaffirmed the critical need to address caregiver mental health as part of the responsive caregiving domain of Nurturing Care, particularly during pregnancy. The lack of measurable iMBC impact on maternal depression could be a reflection of the overall positive impact of having women's groups more broadly. For example, in the group-based parenting intervention in Uganda, women in the intervention group had significantly fewer depressive symptoms over time compared to those in the control groups that did not attend a group-based intervention, even though the intervention groups did not have specific mental health content. While our control C-PrES groups with ECD content did not have a targeted psychological program component, by virtue of meeting, socializing, and supporting each other, the control groups could have had their own positive impact on postpartum mental health. Determining the broader mental health impact of attending socially-oriented women's groups, irrespective of content focused on psychological processes such as cognitive-behavioral therapy like our iMBC intervention, is worth further examination. If similar maternal mental health and child development benefits can be found via a more generic social support women's group, there are implications for the resources needed to implement each. The psychosocial benefits of women's groups are increasingly of interest to the research community (Chomat *et al*., 2019; Preston *et al*., 2019).

Regarding the null findings on the children's socio-emotional development, there are a few areas of possible explanation and/or further inquiry. First, there could have been measurement issues for the ASQ: SE-2 which is designed for screening, not longitudinal assessments, and because this was used in a new context and there may have been issues of cultural applicability, standard cut-off scores would not be appropriate. Alternatively, the socio-emotional development of children under age 2 in this population may tend to be good such that there is minimal room for improvement. The mean item score can range from 0 to 10, with higher being worse, and our sample had a mean of about 2.5–3.0. Finally, the intervention may have had an effect on early stimulation and responsive caregiving behaviors by mothers but the effects on child development were not yet realized given the length of the study.

The implementation challenges for this cognitive-behavioral therapy group-based intervention are noteworthy. This intervention design, in consultation with the GHS, utilized peer mothers with low literacy in the local language and no formal health-care role to deliver the intervention as has been seen in other community-based mental health trials (Singla *et al*., 2017; Verhey *et al*., 2020). This strategy supported the potential sustainability of the intervention but came with literacy challenges for delivering the iMBC content with full fidelity. On a monitoring trip, the training facilitators noted that the illiteracy among about three-quarters of the lead mothers meant that in practice, these lay providers were relying significantly on memory to deliver content. Even though the curriculum materials were a combination of written and picture-based messages, it was still not sufficient to convey and help facilitators remember key components such as reality management and ensuring that participants' experiences were linked with course material explicitly.

The other real-world implementation challenge for this trial was that the intervention was not intended solely as a treatment for mental disorders. Participant eligibility was not based on some pre-defined threshold for mental ill health. CRS as the implementing NGO in close collaboration with the GHS determined that an important purpose of the iMBC intervention was to prevent the onset or further exacerbation of postpartum depression and anxiety and to build skills among women to manage their mental well-being. Our power analysis took this into consideration but as the global mental health field purports to support a prevention-focused strategy, our research tools are still largely based on treating conditions, thus we could be missing important foundational skills building with these community-delivered mental health interventions that could have a realized impact beyond the life of the study.

### Strengths and limitations

This study has a number of strengths and limitations. Data were only collected in two rural districts in northern Ghana and therefore may not be generalized to the general population in Ghana. In addition, while the maternal and child health program from which we sampled was offered to all pregnant women in the study communities, we do not know if we captured the most vulnerable women who did not have the time or resources to participate in the program or in the study. For the child outcomes, we focused on the socio-emotional measure, although we know there are other important development domains not reflected in this analysis. Based on qualitative feedback from stakeholders involved in delivering the program, we know that there were difficulties training and supporting the model mothers to deliver the iMBC content due to literacy issues which means intervention participants may not have received full exposure to the program regardless of attendance issues. Finally, we do not have a true, unexposed control group thus we cannot tease apart the general group effect regardless of educational content delivered in the groups. However, the study strength is the extent of data collection across 32 randomized communities and two local languages that lends important data for community-based programming in Northern Ghana with populations of similar socio-demographic characteristics.

### Implications for research and practice

This intervention did not target women with mental health problems but rather was deployed as a community-based strategy for preventing or reducing depression symptoms among the perinatal population in these rural areas. By virtue of our research-related referrals for mental health care and support to address gender-based violence, we know that participants were open to receiving referrals for more mental health care within the health system although we do not know the rates of follow-up or experiences with the available care in the public sector. Women were less willing to accept our social service referrals for gender-based violence which we know is highly correlated with mental health; however, a number of women did accept the referrals indicating a willingness to engage with formal services (Fisher *et al*., 2012; Laurenzi *et al*., 2020).

Our previous baseline findings from this cohort indicated that poor mental health among our participants was correlated with household hunger, intimate partner violence, and insufficient support from female relatives (Lillie *et al*., 2020). It could be that an integrated maternal mental health and ECD intervention will not reach its intended impact if it does not also directly address these contributing factors to mental ill health. There are increasing calls for the fields of maternal and child health and mental health to take a syndemic approach to programming and more directly address these correlates and pre-disposing factors (Laurenzi *et al*., 2020).

Finally, research should continue to prioritize investigating the effectiveness of community-delivered interventions that address parental mental health and ECD. While lay counselor-delivered mental health interventions are now being prioritized within many low-resource contexts, it is not uncommon that rigorous programs and evaluations are also finding null results when the programs are scaled, indicating a continued knowledge gap for informing countries on effective affordable programming options (Austin *et al*., 2008; Sikander *et al*., 2019). The CRS Ghana team is already exploring ways to train and deliver the iMBC content with a fully pictorially-based iMBC curriculum for peer mothers and even more involvement from husbands/partners in an effort to optimize fidelity. There are new evidence-based resources for ensuring therapeutic competency among non-specialist, lay mental health providers that also could be utilized (Kohrt *et al*., 2015). Efforts to identify effective programming in support of parental mental health and responsive caregiving are critical as these are modifiable pathways that could mitigate suboptimal child development and ensure all children thrive. As the evidence base for targeted ECD interventions grows, we need more real-world evaluations of community-based, lay counselor-led ECD and maternal mental health programs that are embedded in local resources and health and social services systems.

## Supplementary material

For supplementary material accompanying this paper visit http://dx.doi.org/ 10.1017/gmh.2021.15. click here to view supplementary material

## Data

Data are available from the authors upon reasonable request and with the permission of Catholic Relief Services.
