# Supplementary material for: Effect of a lay counselor-delivered integrated maternal mental health and early childhood development group-based intervention in Northern Ghana: a cluster-randomized controlled trial
Source: Glob Ment Health (Camb). 2021 May 26;8:e18. doi: 10.1017/gmh.2021.15 (PMC8157813; doi:10.1017/gmh.2021.15)
Supplement: Supplementary file 1 [file S2054425121000157sup001.docx]

**Supplemental Table S1.** Primary outcomes for maternal mental health (PHQ-9) and child

socio-emotional development (ASQ:SE-2).

| \|  \| **Control** \| **Intervention** \| \| --- \| --- \| --- \| \|  \| **(N = 153)** \| **(N = 221)** \| |
| --- | --- | --- | --- | --- | --- | --- |
| \| **PHQ-9 Score, Baseline** \|  \|  \| \| --- \| --- \| --- \| \| Mean (SD) \| 5.54 (3.82) \| 6.81 (4.21) \| \| N (% Non-missing) \| 153 (100.0%) \| 221 (100.0%) \| \| **PHQ-9 Score, Mini Survey** \|  \|  \| \| Mean (SD) \| 5.00 (3.61) \| 5.92 (4.34) \| \| N (% Non-missing) \| 130 (85.0%) \| 173 (78.3%) \| \| **PHQ-9 Score, Immediate Post-Intervention (Follow-up 1)** \|  \|  \| \| Mean (SD) \| 1.56 (2.80) \| 2.79 (3.81) \| \| N (% Non-missing) \| 131 (85.6%) \| 182 (82.4%) \| \| **PHQ-9 Score, 8-Month Post-Intervention (Follow-up 2)** \|  \|  \| \| Mean (SD) \| 1.40 (2.65) \| 1.96 (2.92) \| \| N (% Non-missing) \| 107 (69.9%) \| 159 (71.9%) \| \| **Mean ASQ:SE-2 Score, Immediate Post-Intervention (Follow-up 1)** \|  \|  \| \| Mean (SD) \| 2.68 (1.32) \| 3.20 (1.56) \| \| N (% Non-missing) \| 130 (85.0%) \| 181 (81.9%) \| \| **Mean ASQ:SE-2 Score, 8-Month Post-Intervention (Follow-up 2)** \|  \|  \| \| Mean (SD) \| 2.34 (0.97) \| 2.51 (1.05) \| \| N (% Non-missing) \| 107 (69.9%) \| 159 (71.9%) \| |

**Supplemental Table S2.** Per-protocol analysis: continuous regression results for PHQ-9 score & ASQ-SE by high attendance intervention group (n=136) versus low attendance + control group (n=207)

| **Maternal Mental Health** | **Predicted mean PHQ-9 in high exposure group** | **Predicted mean PHQ-9 in low exposure group** | **Predicted mean change in PHQ-9 from baseline, high exposure group** | **Predicted mean change in PHQ-9 from baseline, low exposure group** | **Predicted mean difference in change from baseline, high exposure vs. low exposure** |
| --- | --- | --- | --- | --- | --- |
| Baseline | 6.7 (6.0, 7.4) | 6.0 (5.4, 6.6) |  |  |  |
| Short survey | 6.3 (5.6, 7.0) | 5.1 (4.5, 5.7) | -0.4 (-1.1, 0.3) | -0.9 (-1.5, -0.3) | **0.5 (-0.5, 1.4)** |
| Follow-up 1 | 2.8 (2.0, 3.6) | 1.9 (1.2, 2.6) | -3.9 (-4.6, -3.1) | -4.1 (-4.7, -3.4) | **0.2 (-0.8, 1.2)** |
| Follow-up 2 | 1.7 (0.8, 2.6) | 1.8 (1.0, 2.6) | -5.0 (-5.9, -4.1) | -4.2 (-4.9, -3.5) | **-0.8 (-1.9, 0.3)** |
| **Child Socio-emotional development** | **Predicted mean ASQ-SE in high exposure group** | **Predicted mean ASQ-SE in low exposure group** |  |  | **Predicted mean difference, high exposure vs. low exposure** |
| Follow-up 1 | 3.1 (2.4, 3.8) | 2.8 (2.2, 3.4) |  |  | **0.2 (-0.2, 0.5)** |
| Follow-up 2 | 2.4 (1.7, 3.1) | 2.4 (1.8, 3.0) |  |  | **-0.1 (-0.5, 0.2)** |

* High exposure: participants in intervention group who attended more than half or all of the visits

* Low exposure: participants in the control group and participants in the intervention arm received none, unknown or up to about half of the visits

**Supplemental Table S3.** Number of iMBC group sessions attended by participants in treatment arm

|  | Frequency | Percentage | Cumulative percentage |
| --- | --- | --- | --- |
| None | 4 | 1.81 | 1.81 |
| Less than half | 11 | 4.98 | 6.79 |
| About half | 39 | 17.65 | 24.43 |
| More than half | 88 | 39.82 | 64.25 |
| All | 48 | 21.72 | 85.97 |
| Unknown* | 31 | 14.03 | 100.00 |
| Total | 221 | 100.00 |  |

* 28 of the 31 participants with unknown attendance status were lost to follow-up but included in this table as primary analyses were based on intent-to-treat

**Supplemental Table S4.** Illustrative quotes from qualitative analysis regarding main themes

| **Key Informant** | **Illustrative Quotes** |
| --- | --- |
| **iMBC Participants &**  **Lead Mother Facilitators** |  |
| Positive about program | “I think the whole project is an eye opener, very interesting. I enjoyed almost everything about the program, but what stands out is guiding these women to cope with stressors. Observing some of the women, you can see visible signs of depression and it is not that they needed much, but just the course intro, and as the session continued you could see how they control their mood and the improvement in their lives.” |
| Women were able to participate due to familial support | “My husband, my father and mother in-law all gave me the support to participate in the program …I explained to them where I went and the reason for the meeting…they had the understanding of what the program was all about and they said then it is a good program so I should be attending, it will help me.” |
| When asked about iMBC course content, some aspects were deemed challenging such as ‘thought interruption’ and having scheduled ‘worry time.’ | “… the aspect that was difficult for mothers was *thought interruption* whereby they said when women were thinking about something bad, it kept surfacing in their mind. To tell your mind to stop thinking about this is actually a very difficult thing to do.”  “Thought interruption was difficult to follow because you see when you are in a good mood or happy and all of a sudden you get a call or a message of something bad that has happen to your family member or any of your love ones is difficult to overcome that and immediately and engage in a pleasant activity in other to be happy again so for that one I understood but it was difficult practicing it.” |
| **Focus Groups with Husbands** |  |
| Positive about program | “I also know that whenever [CRS] comes and organizes our wives, they talk to them about child delivery and pregnancy in order to help us take good care of our wives.”  “I am also seeing them [CRS] with our wives. It is a happy mood thing and also it concerns our wives’ and children’s health and to take good care of our children so that things will go on well in our houses.” |
| Interested in Engaging in Similar Programs | “If we men too are able to form these groups in addition to the women’s own [groups], it will help improve the living standards in our homes.”  “I would also like to be part of a program like that so I can learn new ideas that will help me care for my children and my family at large.” |
| Biggest parental stressor was lack of money for food, health services, and education. | “As we are in a village like this, one challenge is poverty which always makes us to speak harshly to our wives all because of poverty. Some of us cannot control our anger when we are hit with poverty. Sometimes I will come to the house with poverty anger and will transfer this anger to my wife and children without knowing it.”  “The main challenge here is poverty, because of poverty we can’t afford to buy food from the market to feed our families and last year the crops didn’t do well so no food in most of our houses and I believe this is the reason why our children don’t want to go to school because when they come from school, [there is] no food for them to eat” |
| **Government Stakeholders &**  **Ghana Health Service Supervisors** |  |
| Positive about program, interest in scaling | “I enjoy reading the manual which is about how you can improve your mood and sharing that with the mothers. I can say I like discussion the...discussing the content of the iMBC program with the mothers. That is what I enjoy most.”  “The project is a good initiative that CRS has identified and is investing in it. Personally, I think the government should take over to continue the implementation of the project at the district level now that CRS is wrapping up at the community level. If this is done, it will help more people to also get education on this particular course.” |
| Implementation Challenge: literacy of lead mothers and interpretation of course content in local language | “…explaining things to her [lead mother] for understanding in order for her to also teach the women during a session was difficult…the course content was difficult to translate into local dialect and to relate it in personal life. Some of the English words in the manual…finding a local word which will fit well was always difficult.” |
| Implementation Challenge: regular attendance by participants | “There was lack of motivation, the mothers wanted to get something small (e.g. refreshments, token) at least at the end of the session.”  “The season, like, raining season like this, it’s always difficult to get them.” |
